# Factors influencing the implementation of cardiopulmonary resuscitation among college freshmen: Based on the Theory of Planned Behavior

**DOI:** 10.1371/journal.pone.0337066

**Published:** 2025-12-04

**Authors:** Qinfei Wei, Yu Jiang, Xuemei Lin, Jun Feng, Shan Huang, Feng Wang, Li Fang, Yan Zhang

**Affiliations:** 1 Department of Cardiovascular Medicine, Fuzhou First Hospital Affiliated with Fujian Medical University, Fuzhou, China; 2 Cardiovascular Disease Research Institute of Fuzhou City, Fuzhou, China; 3 Department of Health Screening Centre, Fuzhou First Hospital Affiliated with Fujian Medical University, Fuzhou, China; Universiti Sains Malaysia, MALAYSIA

## Abstract

**Objective:**

Cardiac arrest is a leading cause of global mortality, for which timely cardiopulmonary resuscitation (CPR) is a critical intervention. However, public competence in CPR remains low. Grounded in the Theory of Planned Behavior (TPB), this study investigated the factors influencing CPR implementation among college freshmen by examining their behavioral intention.

**Methods:**

A cross-sectional study was conducted among college freshmen undergoing a basic entrance physical health screening at Fuzhou First General Hospital between September 2 and 9, 2024. Participants were randomly selected to complete an electronic questionnaire, which included a general information sheet and the validated Public Behavior Intention Scale for Performing CPR, adapted with input from emergency medicine experts. Statistical analyses included correlation, regression, and mediation analysis.

**Results:**

Among 4,929 valid questionnaires analyzed, a higher willingness to perform CPR was associated with undergraduate enrollment, good personal and family health status, prior CPR performance, and no history of transient loss of consciousness. Multiple linear regression identified behavioral attitude (β = 0.439), subjective norm (β = 0.272), CPR knowledge (β = 0.027), and perceived behavioral control (β = 0.070) as significant predictors of CPR behavioral intention (all *P* < 0.01). Mediation analysis further revealed that CPR knowledge influenced behavioral intention primarily through its effects on behavioral attitude (79.43% of the total effect), subjective norm (61.31%), and perceived behavioral control (43.67%).

**Conclusion:**

Behavioral attitude serves as the principal pathway through which CPR knowledge translates into intention to act. These findings underscore that targeted CPR training in universities should address both knowledge and psychosocial factors to strengthen students’ willingness to act.

## Introduction

Cardiac arrest (CA) is the cessation of electrical activity of the heart, resulting in the disappearance of circulatory signs [1]. Out-of-hospital cardiac arrest (OHCA) poses a particularly severe and sudden threat to public health [2]. Global statistics estimate that OHCA affects up to 5 million individuals annually, with over 356,000 cases occurring in the United States each year [3,4]. In China, the overall incidence of OHCA is 97.1 per 100,000, accompanied by a notably low 30-day survival rate ranging from 0.2% to 4.6% [5]. The immediate initiation of cardiopulmonary resuscitation (CPR) following OHCA is a critical determinant of patient survival. As the fundamental life-support technique for CA, timely and effective CPR is paramount for improving outcomes and prognosis [6]. Evidence indicates that CPR administered within the “golden 4 minutes” can increase survival rates by 50% to 75% [7]. However, the average response time for specialized emergency medical services (EMS) to reach OHCA patients ranges from 5 to 8 minutes [8]. Therefore, bystanders present before emergency medical services arrive are the best positioned to provide timely CPR.

Despite its proven importance, public proficiency and awareness in CPR remain alarmingly low. College freshmen represent a captive audience at a formative stage, allowing for longitudinal assessment of training retention and serving as the future adult population for the next several years [9]. Some regions, such as the United States and Saudi Arabia, have implemented CPR knowledge and skills as a graduation requirement in certain educational institutions [10]. Surveys indicate that the bystander CPR rate among Chinese adults is less than 1% [11], and a study by Huang et al. found that fewer than 15% of college students had received any CPR training [12].

In light of this training gap, recent scholarly focus has shifted towards understanding the psychological precursors to action. Some studies recommend that CPR education should prioritize fostering the intention to act, making it the central focus of public promotion [13]. The Theory of Planned Behavior (TPB) provides a robust theoretical framework for this endeavor, positing that an individual’s behavioral intention is shaped by three key determinants: behavioral attitude, subjective norm, and perceived behavioral control [14]. The TPB has been extensively and successfully applied to predict a range of health-related intentions among student populations, including smoking, alcohol use, and voluntary blood donation [15,16].

Therefore, this study employs a TPB-guided CPR Behavioral Intention Scale to investigate the determinants of CPR implementation among college freshmen. Crucially, we position CPR knowledge as an antecedent variable and we hypothesize that knowledge exerts its influence by informing and shaping the foundational TPB constructs of attitude, subjective norm, and perceived behavioral control. This conceptual integration aligns with the Knowledge-Attitude-Practice (KAP) model [17], illustrating a pathway from cognitive awareness to behavioral intention. The findings are expected to provide insights that may inform future educational strategies on CPR training in universities.

## Methods

### Study design and participants

This cross-sectional study was conducted among college freshmen presenting for the basic entrance physical screenings at Fuzhou First Hospital between September 2 and 9, 2024. A simple random sampling method was used to recruit participants daily from the master list of scheduled students. Eligibility required participants to be college freshmen of any academic major, undergoing the standard physical screening, and providing voluntary informed consent. All participants completed an anonymous, self-administered electronic questionnaire via the Questionnaire Star platform (Wenjuanxing, https://www.wjx.cn/).

To ensure data quality, questionnaires were excluded based on pre-defined criteria for invalid responses, including incomplete questionnaires, internal response inconsistencies, or patterns of straight-lining (full exclusion criteria in S1 Appendix). From an initial 5,000 collected questionnaires, 71 were excluded following these criteria, yielding 4,929 valid responses for analysis (effective response rate: 98.58%).

This study complied with the requirements of medical ethics and was approved by the Ethics Committee of the Fuzhou First General Hospital (Approval No: 202411003). All participants provided informed consent.

### The questionnaire

A general information questionnaire was developed based on literature review and the study objectives. It included items on sex, birthplace, ethnicity, academic qualification, major, personal and family health status, cohabitation with elderly relatives, prior CPR performance, previous CPR training, and history of transient loss of consciousness.

The CPR questionnaire was adapted from the validated “Development and psychometric test of the Public Behavior Intention Scale for Performing Cardiopulmonary Resuscitation” designed by Zheng et al. [18]. To enhance conciseness and participant engagement for the large-scale health screening context, a subset of items was selected from the original instrument. This process was guided by a panel of five experts in emergency medicine and nursing. To ensure content validity, the expert panel evaluated the relevance and clarity of the selected items in measuring the intended TPB constructs within the context of college freshmen. The selection prioritized: (1) items with high factor loadings from the original study to preserve structural validity; (2) comprehensive coverage of each theoretical construct; and (3) clarity and conciseness to minimize participant burden.

The final adapted instrument comprised five subscales: CPR knowledge, behavioral attitude, subjective norm, perceived behavioral control, and behavioral intention. The knowledge subscale included 8 items, each scored 1 for a correct answer and 0 for an incorrect answer, for a total of 0–8 points, with higher scores indicating better knowledge. The remaining four subscales used a 5-point Likert scale (1 = strongly disagree, 2 = disagree, 3 = neutral, 4 = agree, and 5 = strongly agree). Behavioral attitude was measured with 5 items (total score 5–25), subjective norm with 6 items (total score 6–30), perceived behavioral control with 8 items (total score 8–40), and behavioral intention with 3 items (total score 3–15). For each subscale, higher scores indicate more positive or favourable CPR-related perceptions.

A pre-survey with 150 college freshmen was conducted to test the adapted questionnaire, demonstrating good reliability (Cronbach’s alpha = 0.83). As it employed the final questionnaire and identical protocol, these data were included in the final analysis to increase statistical power. The full list of items used in this study is provided in S2 Appendix.

### Quality control

Rigorous quality control measures were implemented throughout the data collection process. The survey was conducted anonymously, with technical limitations ensuring each mobile device could submit only once, thereby preventing duplicate entries. Following data retrieval, all responses underwent a manual double-checking procedure. Any questionnaire identified as invalid based on the pre-defined criteria in S1 Appendix was excluded to uphold data integrity. Participation was contingent upon informed consent; the survey platform was configured to automatically exit if a user declined, ensuring that all analyzed data originated from consenting individuals.

### Statistical analysis

Data analysis was performed using SPSS Statistics (Version 29.0) and R software (Version 4.0). Continuous variables, all of which significantly deviated from a normal distribution per the Shapiro-Wilk test (*P* < 0.05), are presented as median and interquartile range (IQR). Categorical data are summarized using frequencies and percentages (%). Group comparisons for demographic characteristics were conducted using the Mann-Whitney U test or the Kruskal-Wallis H test.

Associations among the key study variables (CPR knowledge, behavioral attitude, subjective norm, perceived behavioral control, and behavioral intention) were examined using Spearman correlation analysis. After adjustment for covariates, univariable linear regression and multivariable linear regression were used to investigate the factors influencing CPR behavioral intention, presented as regression coefficient (β) with 95% confidence intervals (CI). Subgroup analyses were performed to investigate potential interactions across various demographic factors.

To delineate the mediating roles of the TPB constructs, a series of mediation analyses were conducted using the R ‘mediation’ package. We tested three separate models, each specifying CPR knowledge as the independent variable, behavioral intention as the dependent variable, and one of the three TPB constructs (behavioral attitude, subjective norm, or perceived behavioral control) as the mediator. This approach allowed for the isolation of the unique indirect effect of each mediator. The analyses utilized a quasi-Bayesian Monte Carlo simulation method with 1000 iterations. The direct effect denotes the association between CPR knowledge and intention in the absence of the mediator, while the indirect effect represents the pathway through the mediator. A two-tailed p-value of < 0.05 was considered statistically significant for all tests.

## Results

### Participant characteristics

The socio-demographic characteristics of the participants were summarized in Table 1. Briefly, the majority of participants were from rural areas (67.6%) and were male (56.6%). Most participants were enrolled in undergraduate programs (66.8%) and were science majors (67.8%). Over half reported no prior CPR performance (97.4%) or training (58.4%), and most had no history of transient loss of consciousness (85.3%).

**Table 1:** General information and CPR-related information of the participants (*N* = 4929).

Variable		*N* (%)		CPR knowledge	Behavioral attitude	Subjective norm	Perceived behavioral control	Behavioral intention
**Sex**								
	Male	2792(56.6)		4.00 (2.00, 5.00)	23.00 (20.00, 25.00)	24.00 (23.00, 30.00)	32.00 (24.00, 40.00)	14.00 (12.00, 15.00)
	Female	2137(43.3)		4.00 (3.00, 6.00)	23.00 (20.00, 25.00)	24.00 (21.00, 30.00)	30.00 (24.00, 36.00)	13.00 (12.00, 15.00)
			*P*	<0.001	0.910	<0.001	<0.001	0.071
**Birthplace**								
	Urban	1596(32.3)		4.00 (3.00, 6.00)	23.00 (20.00, 25.00)	24.00 (23.00, 30.00)	32.00 (24.00, 40.00)	14.00 (12.00, 15.00)
	Countryside	3333(67.6)		4.00 (3.00, 5.00)	22.00 (20.00, 25.00)	24.00 (22.00, 30.00)	31.00 (24.00, 40.00)	14.00 (12.00, 15.00)
			*P*	0.012	<0.001	<0.001	<0.001	0.385
**Ethnicity**								
	Han ethnic group	4788(97.1)		4.00 (3.00, 5.00)	23.00 (20.00, 25.00)	24.00 (22.00, 30.00)	32.00 (24.00, 40.00)	14.00 (12.00, 15.00)
	Other ethnic groups	141(2.8)		4.00 (2.00, 6.00)	22.00 (20.00, 25.00)	24.00 (22.00, 30.00)	32.00 (24.00, 40.00)	12.50 (12.00, 15.00)
			*P*	0.824	0.395	0.876	0.932	0.190
**Academic qualification**								
	Post-secondary	1633(33.1)		4.00 (2.00, 5.00)	22.00 (20.00, 25.00)	24.00 (22.00, 30.00)	32.00 (24.00, 40.00)	13.00 (12.00, 15.00)
	Undergraduate and above	3296(66.8)		4.00 (3.00, 5.00)	23.00 (20.00, 25.00)	24.00 (22.00, 30.00)	30.00 (24.00, 40.00)	14.00 (12.00, 15.00)
			*P*	<0.001	0.063	0.072	<0.001	0.005
**Major**								
	Humanities	1567(31.7)		4.00 (3.00, 6.00)	23.00 (20.00, 25.00)	24.00 (22.00, 30.00)	32.00 (24.00, 40.00)	14.00 (12.00, 15.00)
	Science	3344(67.8)		4.00 (3.00, 5.00)	22.00 (20.00, 25.00)	24.00 (22.00, 30.00)	32.00 (24.00, 40.00)	14.00 (12.00, 15.00)
			*P*	0.291	0.005	0.797	0.074	0.614
**Personal health status**								
	Poor	74(1.4)		4.00 (3.00, 5.00)	23.50 (19.00, 25.00)	24.00 (18.00, 30.00)	29.50 (24.00, 40.00)	12.00 (9.00, 15.00)
	Usual	1692(34.3)		4.00 (2.00, 5.00)	20.50 (19.00, 25.00)	24.00 (19.00, 30.00)	26.00 (24.00, 32.00)	12.00 (11.00, 15.00)
	Good	3163(64.1)		4.00 (3.00, 6.00)	24.00 (20.00, 25.00)	26.00 (24.00, 30.00)	32.00 (24.00, 40.00)	15.00 (12.00, 15.00)
			*P*	0.002	<0.001	<0.001	<0.001	<0.001
**Family health status**								
	Poor	121(2.3)		4.00 (3.00, 5.00)	22.00 (18.00, 25.00)	24.00 (19.00, 30.00)	28.00 (24.00, 37.50)	13.00 (11.00, 15.00)
	Usual	1484(30.1)		4.00 (2.00, 5.00)	20.00 (19.00, 25.00)	24.00 (19.00, 30.00)	27.00 (24.00, 32.00)	12.00 (11.00, 15.00)
	Good	3324(67.4)		4.00 (3.00, 6.00)	24.00 (20.00, 25.00)	25.00 (23.00, 30.00)	32.00 (24.00, 40.00)	15.00 (12.00, 15.00)
			*P*	0.021	<0.001	<0.001	<0.001	<0.001
**Cohabitation with elderly relatives**								
	Yes	1427(28.9)		4.00 (3.00, 5.00)	22.00 (20.00, 25.00)	24.00 (22.00, 30.00)	31.00 (24.00, 40.00)	14.00 (12.00, 15.00)
	No	3502(71.0)		4.00 (3.00, 5.00)	23.00 (20.00, 25.00)	24.00 (22.00, 30.00)	32.00 (24.00, 40.00)	14.00 (12.00, 15.00)
			*P*	0.846	0.518	0.287	0.143	0.885
**Prior CPR performance**								
	Yes	127(2.5)		5.00 (4.00, 7.00)	25.00 (20.00, 25.00)	30.00 (24.00, 30.00)	39.00 (31.00, 40.00)	15.00 (12.00, 15.00)
	No	4802(97.4)		4.00 (3.00, 5.00)	22.00 (20.00, 25.00)	24.00 (22.00, 30.00)	32.00 (24.00, 40.00)	14.00 (12.00, 15.00)
			*P*	<0.001	<0.001	<0.001	<0.001	0.002
**Previous CPR training**								
	Yes	2048(41.5)		5.00 (3.00, 6.00)	23.00 (20.00, 25.00)	24.00 (22.00, 30.00)	32.00 (24.00, 40.00)	14.00 (12.00, 15.00)
	No	2881(58.4)		3.00 (2.00, 5.00)	22.00 (20.00, 25.00)	24.00 (22.00, 30.00)	32.00 (24.00, 40.00)	13.00 (12.00, 15.00)
			*P*	<0.001	0.005	0.781	0.117	0.190
**History of transient loss of consciousness**								
	Yes	721(14.6)		4.00 (3.00, 5.00)	22.00 (20.00, 25.00)	24.00 (21.00, 30.00)	28.00 (24.00, 32.00)	13.00 (12.00, 15.00)
	No	4208(85.3)		4.00 (3.00, 5.00)	23.00 (20.00, 25.00)	24.00 (22.00, 30.00)	32.00 (24.00, 40.00)	14.00 (12.00, 15.00)
			*P*	0.277	0.021	<0.001	<0.001	<0.001

Note: Data are presented as absolute numbers (percentages) for categorical variables and median (IQR) for continuous variables with skewed data. Mann-Whitney U test and Kruskal-Wallis H test for skewed continuous variables. Abbreviations: CPR, cardiopulmonary resuscitation.

### Univariate analysis of scale scores

Nonparametric tests showed that participants who were female, urban, undergraduate, reported good personal or family health status had performed CPR before, or had received prior CPR training demonstrated better CPR knowledge (all *P* < 0.05). More positive behavioral attitudes were observed among participants who were from urban areas, undergraduate students, humanities majors, in good personal and family health, had prior CPR experience or training, or had no history of fainting (all *P* < 0.05). Higher scores for subjective norms and perceived behavioral control were associated with being male, from urban areas, in good personal and family health, having performed CPR previously, or having no syncope history (all *P* < 0.05). Furthermore, participants who were undergraduates, in good personal and family health, with prior CPR performance, or no syncope history reported a significantly higher willingness to perform CPR in an out-of-hospital cardiac arrest scenario (all *P* < 0.05).

### Correlations among TPB constructs and CPR knowledge

Spearman’s correlation analysis revealed significant positive correlations between CPR behavioral intention and all other measured variables. As detailed in Table 2, behavioral intention was most strongly correlated with behavioral attitude (r = 0.713, *P* < 0.05) and subjective norm (r = 0.700, *P* < 0.05), followed by perceived behavioral control (r = 0.575, *P* < 0.05). A weaker, though statistically significant, positive correlation was found between CPR knowledge and behavioral intention (r = 0.159, *P* < 0.05).

**Table 2:** Spearman correlation matrix among the main study variables (*N* = 4929).

Items	CPR knowledge	Behavioral attitude	Subjective norm	Perceived behavioral control	Behavioral intention
**CPR knowledge**	–				
**Behavioral attitude**	0.172	–			
**Subjective norm**	0.126	0.763	–		
**Perceived behavioral control**	0.103	0.611	0.745	–	
**Behavioral intention**	0.159	0.713	0.700	0.575	–

Note: All correlation coefficients are statistically significant at *P* < 0.001. Abbreviations: CPR, cardiopulmonary resuscitation.

### Regression and subgroup analyses of behavioral intention predictors

**Fig 1:**
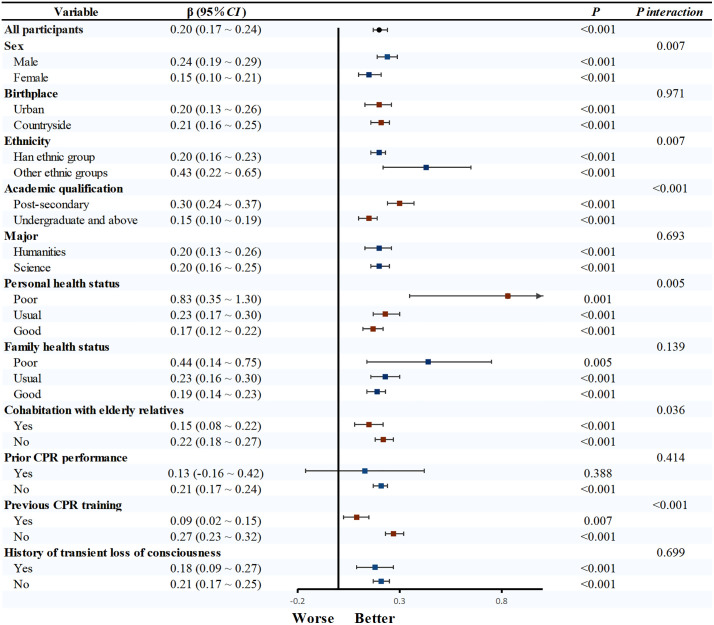
Subgroup analysis of CPR knowledge and CPR behavioral intention. Note: After adjusting for all factors except the study factors, linear regression was used to obtain effect values. Abbreviations: CPR, cardiopulmonary resuscitation; CI, confidence interval.

**Fig 2:**
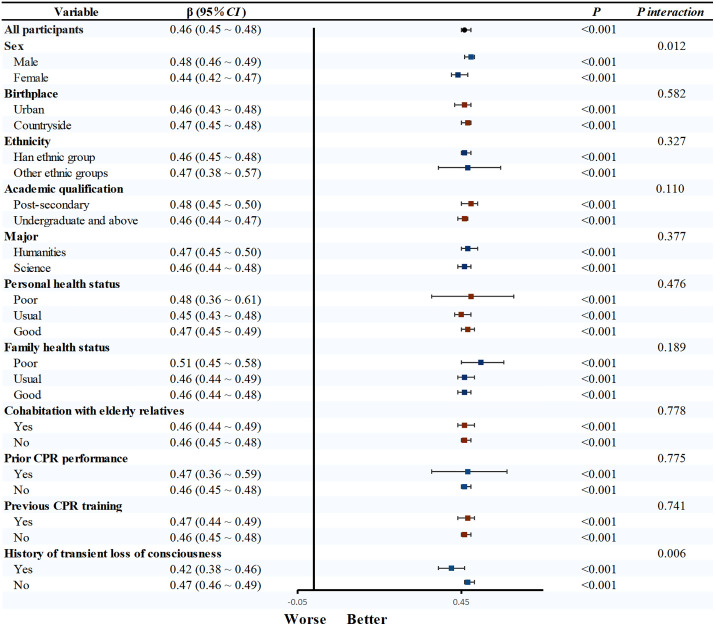
Subgroup analysis of behavioral attitude and CPR behavioral intention. Note: After adjusting for all factors except the study factors, linear regression was used to obtain effect values. Abbreviations: CPR, cardiopulmonary resuscitation; CI, confidence interval.

**Fig 3:**
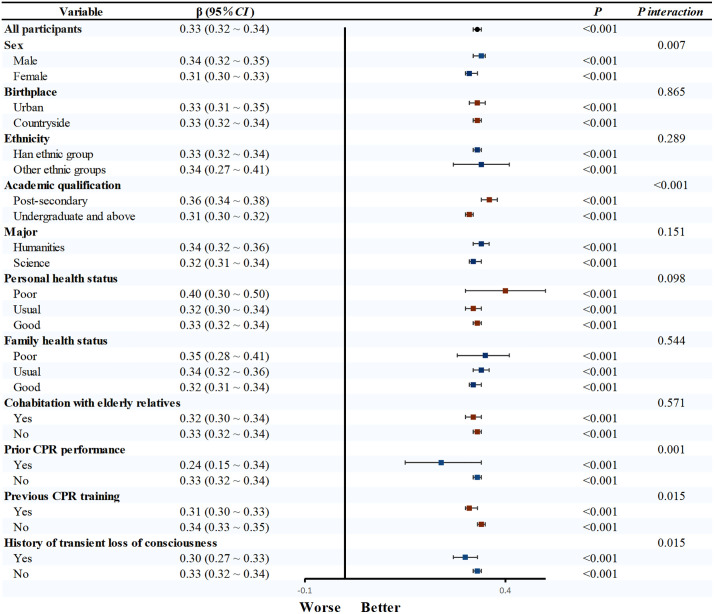
Subgroup analysis of subjective norm and CPR behavioral intention. Note: After adjusting for all factors except the study factors, linear regression was used to obtain effect values. Abbreviations: CPR, cardiopulmonary resuscitation; CI, confidence interval.

**Fig 4:**
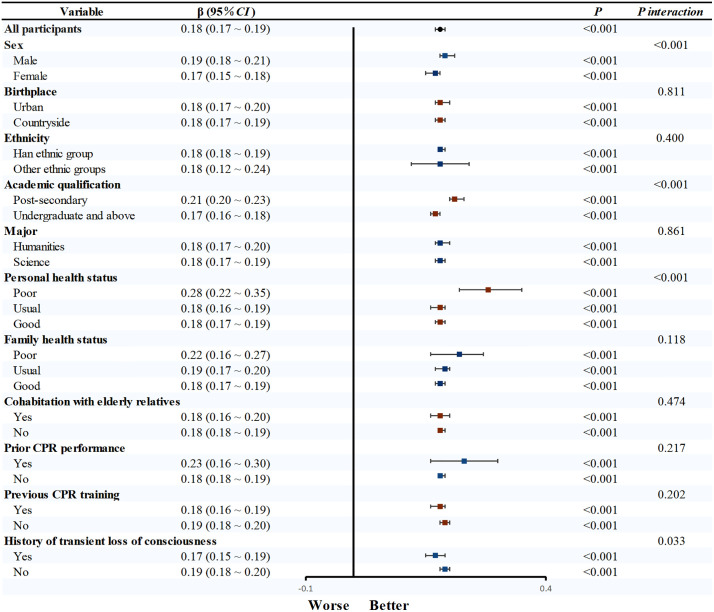
Subgroup analysis of perceived behavioral control and CPR behavioral intention. Note: After adjusting for all factors except the study factors, linear regression was used to obtain effect values. Abbreviations: CPR, cardiopulmonary resuscitation; CI, confidence interval.

Univariable linear regression confirmed CPR knowledge, behavioral attitude, subjective norm, and perceived behavioral control as significant determinants of CPR behavioral intention (*P* < 0.05). Subsequent subgroup analyses revealed that the strength of the association between CPR knowledge and behavioral intention varied significantly across different demographics. Specifically, this relationship was more pronounced in males (β = 0.24, 95% CI: 0.19–0.29), other ethnic groups (β = 0.43, 95% CI: 0.22–0.65), post-secondary (β = 0.30, 95% CI: 0.24–0.37), poor health status (β = 0.83, 95%CI: 0.35–1.30), not living with the elderly (β = 0.22, 95%CI: 0.18–0.27), and those without prior CPR training (β = 0.27, 95%CI: 0.23–0.32) (all *P*_interaction_ < 0.05; Fig 1). Similarly, the influence of behavioral attitude on intention was significantly stronger in males (β = 0.48, 95% CI: 0.46–0.49) and those with no fainting history (β = 0.47, 95% CI: 0.46–0.49, Fig 2). Significant interaction effects were also observed for subjective norm and perceived behavioral control across various demographic factors (all *P* < 0.05, Figs 3 and 4).

### Multivariable linear regression analysis

A multiple linear regression was conducted to predict CPR behavioral intention from CPR knowledge, behavioral attitude, subjective norm, and perceived behavioral control. The overall model was statistically significant and explained 52.5% of the variance in intention (Adjusted R² = 0.525, F(4, 4924) = 1364.162, *P* < 0.001). All variance inflation factors (VIF) were below 5, indicating no concerns regarding multicollinearity. As shown in Table 3, behavioral attitude was the strongest predictor (β = 0.439, *P* < 0.001), followed by subjective norm (β = 0.272, *P* < 0.001), with CPR knowledge (β = 0.027, *P* = 0.007) and perceived behavioral control (β = 0.070, *P* < 0.001) contributing modest but significant effects.

**Table 3:** Multiple linear regression analysis of influencing CPR behavioral intention (*N* = 4929).

Variable	Unstandardized Coefficients (B)	Standard error (SE)	Standardized Coefficients (β)	*t*	*P*	*VIF*
**Constant**	2.217	0.149		14.829	<0.001	
**CPR knowledge**	0.035	0.013	0.027	2.715	0.007	1.039
**Behavioral attitude**	0.296	0.010	0.439	28.763	<0.001	2.414
**Subjective norm**	0.136	0.009	0.272	15.222	<0.001	3.325
**Perceived behavioral control**	0.024	0.005	0.070	4.841	<0.001	2.196

Note: The regression model was statistically significant, F(4, 4924) = 1364.162, *P* < 0.001. The model explained 52.5% of the variance in behavioral intention (Adjusted R² = 0.525). All VIF values were well below 5, indicating no substantial multicollinearity. Abbreviations: CPR, cardiopulmonary resuscitation; VIF, variance inflation factor.

### Mediation analysis

**Fig 5:**
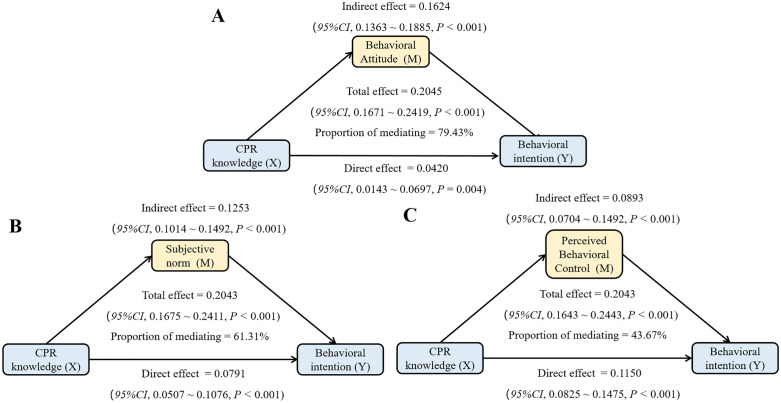
The mediating role of behavioral attitude, subjective norm and perceived behavioral control. Note: We adjusted for all factors except research factors. Figure A shows the association between CPR knowledge and CPR behavioral intention mediated by behavioral attitude; Figure B shows the association between CPR knowledge and CPR behavioral intention mediated by subjective norms; Figure C shows the association between CPR knowledge and CPR behavioral intention mediated by perceived behavioral control. Abbreviations: CPR, cardiopulmonary resuscitation; CI, confidence interval.

The mediation analysis, illustrated in Fig 5, assessed whether the TPB constructs mediated the relationship between CPR knowledge and behavioral intention. The results indicated significant indirect effects through all three proposed mediators. Specifically, behavioral attitude accounted for the largest proportion of the total effect (79.43%), followed by subjective norm (61.31%) and perceived behavioral control (43.67%) (all *P* < 0.001).

## Discussion

This study employed the TPB to investigate the factors influencing the behavioral intention toward CPR among a large sample of Chinese university freshmen. Relative to other TPB studies on college students [19–23], this study conducted a cross-sectional survey of 4929 college freshmen’s willingness to perform CPR and their influencing factors, which shows that the large sample size of the data is one of the strengths of this study. Our findings confirm the utility of the TPB framework in this context, where college freshmen’s knowledge of CPR, behavioral attitude, subjective norm, and perceived behavioral control significantly influence their intention to perform CPR. A pivotal contribution of this study lies in elucidating the mediating pathways, revealing that the influence of CPR knowledge on intention is predominantly channeled through these TPB constructs, with attitude serving as the primary conduit.

The observed positive association between higher education levels and increased willingness to perform CPR is consistent with existing literature, which reports greater intent among more educated groups compared to high school students [24], among whom willingness often falls below 50% [25]. Furthermore, people with better mental health are more willing to help others [26]. In addition, self-rated health was found to be negatively correlated with depression [27]. This could explain the fact that people with good personal health and family health in this study may be performing well psychologically and thus be more willing to perform CPR to rescue a critically ill person. Finally, a history of transient loss of consciousness was associated with lower CPR intention. This may be explained by a heightened state of fear or anxiety in emergencies, which can distract from the focus required to perform CPR [28]. Thus, an individual’s capacity to maintain composure appears to be a critical, though often overlooked, component of CPR behavioral intention.

In addition, several interesting points were found in our findings. First, females demonstrated greater CPR knowledge, potentially due to gender-role socialization that emphasizes health and caregiving [29]. Conversely, males reported higher subjective norms and perceived behavioral control, possibly reflecting greater social expectations for them to act and confidence derived from physical strength advantages. Second, the 127 participants with prior CPR performance exhibited markedly high scores across all TPB constructs, with perceived behavioral control being the most prominent. This suggests that the hands-on act of performing CPR significantly enhances an individual’s confidence in their ability to act effectively. This is corroborated by the findings of Jia et al. [28] and Lu et al. [30], indicating that practical experience is a powerful driver of the psychological factors that underpin behavioral intention, likely by solidifying skills, affirming competence, and internalizing the value of intervention.

In the regression analysis, CPR behavioral attitude had the most significant effect on CPR behavioral intention. College students have a positive attitude toward CPR and believe that CPR is necessary in emergencies, and this positive attitude can significantly increase their willingness to perform CPR. One study showed that the vast majority of rescuers were willing to perform CPR again, and this was because they believed that performing CPR was a positive experience [31]. When an individual holds a positive attitude toward a behavior, the likelihood of their behavioral intention and actual behavior increases. Subjective norm was also a significant predictor, highlighting the powerful role of perceived social expectations. This influence is likely amplified in collectivist cultures like China, where social harmony and fulfilling group expectations are highly valued. Our results closely align with those of Magid et al. [32], who similarly identified attitude and subjective norm as principal drivers of CPR intention in their student population. The convergence of these findings across different contexts reinforces the robustness of these TPB constructs, while our larger sample size affords greater precision to these estimates. Subgroup analysis further revealed that the impact of CPR knowledge on behavioral intention was more pronounced among males and individuals who had not previously received CPR training. Particularly for untrained individuals, knowledge served as the primary and most direct catalyst, directly filling critical cognitive gaps and substantially enhancing behavioral intention.

Positioning CPR knowledge as a critical antecedent variable, our findings align with the KAP model, which posits that knowledge serves as the foundational step toward behavioral change. This is consistent with the results of Karuthan et al., who identified a significant association between CPR knowledge and the willingness to act [33]. The primary vehicle for disseminating this knowledge is formal CPR training, which is essential for building a community capable of supporting emergency medical services [29]. However, our data reveal a substantial training gap, with only 41.5% of the surveyed freshmen having received such instruction. This deficit is particularly consequential given robust evidence that trained individuals, irrespective of their professional background, possess significantly higher CPR knowledge than their untrained counterparts [34,35]. Moreover, the retention of this knowledge and corresponding skills is contingent upon regular refresher training, as proficiency has been shown to decline over time even among healthcare providers [36]. Finally, the high mediation effect of attitude indicates that knowledge primarily enhances intention by shaping positive attitudes toward CPR, underscoring the need for educational programs to foster positive beliefs and outcome expectations, not merely factual knowledge. Therefore, moving beyond the mere transmission of facts, we recommend that interventions designed to improve CPR implementation among college students adopt an integrated strategy: (1) Institute regular and mandatory CPR training to establish and maintain a robust baseline of knowledge; (2) Explicitly target attitude formation within educational content, emphasizing the lifesaving potential of CPR, the rescuer’s vital role, and the positive outcomes of intervention to cultivate a strong sense of efficacy and responsibility; (3) Strengthen perceived behavioral control through high-fidelity simulation and repetitive practice, thereby building the self-efficacy necessary to translate intention into action under stressful conditions.

This study’s key contributions are its large, focused sample and the quantification of mediation pathways. These findings precisely demonstrate that knowledge enhances CPR intention mainly by shaping attitudes and norms, informing targeted educational strategies. Notwithstanding these insights, several limitations warrant consideration. First, the cross-sectional design captures intention rather than actual behavior. Second, self-report bias may lead to an overestimation of positive attitudes and intentions. Third, although this study involved a large sample size, it remains a single-center study conducted in a city in China. Future plans include expanding it into a multicenter study.

## Conclusion

This study examined factors influencing college freshmen’s intention to perform CPR using the Theory of Planned Behaviour. Behavioral attitude and subjective norm emerged as the strongest predictors of intention, with knowledge and perceived behavioral control also contributing significantly. Mediation analysis suggested that the effect of knowledge on intention operates largely through its influence on attitudes and norms. These findings support the importance of integrating CPR training into university programmes, with attention not only to knowledge but also to fostering positive attitudes and supportive social norms around CPR.

## Supporting information

S1 Appendix Detailed breakdown of excluded questionnaires (*N* = 71). (PDF)

S2 Appendix The questionnaire. (PDF)
