# Supplementary material for: Factors influencing the implementation of cardiopulmonary resuscitation among college freshmen: Based on the Theory of Planned Behavior
Source: PLoS One. 2025 Dec 4;20(12):e0337066. doi: 10.1371/journal.pone.0337066 (PMC12677528; doi:10.1371/journal.pone.0337066)
Supplement: S1 Appendix — (PDF) [file pone.0337066.s001.pdf]

S1 Appendix. Detailed breakdown of excluded questionnaires ($N = 71$).

Exclusion criteria	Operational Definition	Number (%)
**Incomplete questionnaires**	Questionnaires with more than 10% of items missing across the entire survey, or any missing data within the critical CPR behavioral intention subscale, were excluded.	38 (53.52%)
**Internal response inconsistencies**	Failed more than one embedded logic check. Responses that were not logically congruent were flagged, and participants with more than one such inconsistency were excluded.	4 (5.63%)
**Patterns of straight-lining**	Questionnaires were excluded if they exhibited a fixed response pattern for over 80% of the items within one or more of the multi-item Likert scale subscales, coupled with excessively short response times.	29 (40.85%)
**Total**		71 (100%)
