# Supplementary material for: Factors influencing the implementation of cardiopulmonary resuscitation among college freshmen: Based on the Theory of Planned Behavior
Source: PLoS One. 2025 Dec 4;20(12):e0337066. doi: 10.1371/journal.pone.0337066 (PMC12677528; doi:10.1371/journal.pone.0337066)
Supplement: S2 Appendix — (PDF) [file pone.0337066.s002.pdf]

S2 Appendix. The questionnaire.

**Survey on College Students' Willingness to Perform CPR**	
**Dear Classmate,**<br>You are cordially invited to participate in this research survey, which aims to investigate the willingness of Chinese university students to perform bystander cardiopulmonary resuscitation (CPR). Your valuable input will provide critical scientific evidence for developing strategies to promote CPR awareness and training within the student population.<br>This questionnaire will take approximately 5-10 minutes to complete. We assure you that all responses will be kept strictly anonymous and confidential. Please respond to each item based on your genuine perceptions and circumstances.<br>Your participation is highly valued and will contribute significantly to this important initiative. Thank you for your time and support.	
Would you like to participate in this survey? (If you choose "Yes," please continue; if you choose "No," you may exit the survey)	□ Yes<br>□ No
**Section One: Personal Information (Please mark "√" next to the selected answer or fill in the appropriate blank with your real response. For multiple-choice questions with explanations, please select as applicable.)**	
1. Gender	□ Male<br>□ Female
2. Birthplace	□ Urban<br>□ Countryside
3. Ethnicity	□ Han ethnic group<br>□ Other ethnic groups
4. Academic qualifications	□ Post-secondary<br>□ Undergraduate and above
5. Major<br>"Humanities" refers to fields including arts, social sciences, and business.	□ Humanities<br>□ Science

"Science" refers to fields including engineering, medicine, and natural sciences.	
6. Personal health status<br>"Poor" refers to being in poor physical condition or having specific illnesses.<br>"Usual" refers to being in generally good health but have some minor ailments.<br>"Good" indicates that one is free from illness and in a healthy or good physical condition.	□ Poor<br>□ Usual<br>□ Good
7. Family health status<br>"Poor" refers to family members being in poor physical condition or having specific illnesses.<br>"Usual" means that family members are generally in good shape but have some minor ailments.<br>"Good" indicates that all family members are free from illness and in a healthy or good physical condition.	□ Poor<br>□ Usual<br>□ Good
8. Cohabitation with elderly relatives<br>(Living with individuals aged 65 years or older.)	□ Yes<br>□ No
9. Prior CPR performance<br>(Actual, hands-on delivery of CPR in a real-life emergency situation.)	□ Yes<br>□ No
10. Previous CPR training<br>(Formal instruction in CPR techniques, regardless of the setting (e.g., course, workshop).)	□ Yes<br>□ No
11. History of transient loss of consciousness<br>(A prior episode of fainting (syncope) or a temporary blackout.)	□ Yes<br>□ No
**Section Two: CPR knowledge (Please mark "√" in the appropriate box for each item based on your own understanding. Only one answer is allowed for each item.)**	
1. What is the "golden window" for initiating CPR after a person experiences a sudden cessation of breathing and heartbeat?	□ Within 4 minutes.<br>□ Within 10 minutes.<br>□ Unsure.
2. What is the correct method to assess for unresponsiveness in a suspected cardiac arrest	□ Shout loudly and tap the shoulder.<br>□ Shout loudly and gently shake the

victim?	shoulders.<br>□ Shout loudly and slap the cheeks.<br>□ Unsure.
3. When performing external cardiac compression, the patient should lie on a ____?	□ Shout loudly and tap the shoulder.<br>□ Shout loudly and gently shake the shoulders.<br>□ Shout loudly and slap the cheeks.<br>□ Unsure.
4. What is the correct position of chest compressions in adult CPR?	□ Median nipple line in the chest.<br>□ the lower 1/3 of the sternum.<br>□ 5 centimeters below the left nipple.<br>□ Unsure.
5. What is the proper depth of chest compressions in adult CPR?	□ 2-3 centimeters.<br>□ 3-4 centimeters.<br>□ 5-6 centimeters.<br>□ Unsure.
6. What is the proper frequency of chest compressions in adult CPR?	□ 60-80 times per minute.<br>□ 80-100 times per minute.<br>□ 100-120 times per minute.<br>□ Unsure.
7. What is the correct procedure for CPR?<br>A. Breathing.<br>B. Airway.<br>C. Compression.	□ ABC.<br>□ BCA.<br>□ CBA.<br>□ Unsure.
8. What is the pressure-to-air ratio in adult CPR?	□ 15:1.<br>□ 30:2.<br>□ 30:1.<br>□ Unsure.

**Section Three: Behavioural attitude (Please mark "√" in the appropriate box for each item based on your own understanding. Only one answer is allowed for each item.)**	
I believe/I know/I understand that performing CPR	
1. may save the lives of patients experiencing cardiac arrest.	□ Strongly disagree.<br>□ Disagree.<br>□ Neutral.<br>□ Agree.<br>□ Strongly agree.
2. may not always be effective, but one should still actively administer it.	□ Strongly disagree.<br>□ Disagree.<br>□ Neutral.<br>□ Agree.<br>□ Strongly agree.
3. may cause secondary injury to the patient's body, but compared to saving a life, this is acceptable.	□ Strongly disagree.<br>□ Disagree.<br>□ Neutral.<br>□ Agree.<br>□ Strongly agree.
4. to save another person's life is a meaningful act for society.	□ Strongly disagree.<br>□ Disagree.<br>□ Neutral.<br>□ Agree.<br>□ Strongly agree.
5. to save someone's life is a source of pride and honor for me.	□ Strongly disagree.<br>□ Disagree.<br>□ Neutral.<br>□ Agree.<br>□ Strongly agree.

**Section Four: Subjective norm (Please mark "√" in the appropriate box for each item based on your own understanding. Only one answer is allowed for each item.)**	
If I have received CPR training and happen to be present at the scene of an out-of-hospital cardiac arrest	
1. my family and friends believe I should perform CPR on the patient.	□ Strongly disagree.<br>□ Disagree.<br>□ Neutral.<br>□ Agree.<br>□ Strongly agree.
2. the patient and their family members believe I should perform CPR on the patient.	□ Strongly disagree.<br>□ Disagree.<br>□ Neutral.<br>□ Agree.<br>□ Strongly agree.
3. mainstream societal values dictate that bystanders should perform CPR on the patient.	□ Strongly disagree.<br>□ Disagree.<br>□ Neutral.<br>□ Agree.<br>□ Strongly agree.
4. I am willing to perform CPR on the patient in accordance with the wishes of their family and friends.	□ Strongly disagree.<br>□ Disagree.<br>□ Neutral.<br>□ Agree.<br>□ Strongly agree.
5. I am willing to perform CPR on the patient in accordance with the wishes of the patient and their family.	□ Strongly disagree.<br>□ Disagree.<br>□ Neutral.<br>□ Agree.

	□ Strongly agree.
6. I am willing to perform CPR on the patient in accordance with the expectations of mainstream societal values.	□ Strongly disagree.<br>□ Disagree.<br>□ Neutral.<br>□ Agree.<br>□ Strongly agree.
**Section Five: Perceived behavioural control (Please mark "√" in the appropriate box for each item based on your own understanding. Only one answer is allowed for each item.)**	
If I have received CPR training and happen to be present at the scene of an out-of-hospital cardiac arrest	
1. I am confident in my ability to perform CPR.	□ Strongly disagree.<br>□ Disagree.<br>□ Neutral.<br>□ Agree.<br>□ Strongly agree.
2. I believe I could accurately determine whether the patient had suffered cardiac arrest.	□ Strongly disagree.<br>□ Disagree.<br>□ Neutral.<br>□ Agree.<br>□ Strongly agree.
3. I believe I could effectively perform chest compressions.	□ Strongly disagree.<br>□ Disagree.<br>□ Neutral.<br>□ Agree.<br>□ Strongly agree.
4. I believe I could effectively perform rescue breathing.	□ Strongly disagree.<br>□ Disagree.<br>□ Neutral.

	□ Agree.<br>□ Strongly agree.
5. I believe I could effectively administer automated external defibrillation using an AED (if available).	□ Strongly disagree.<br>□ Disagree.<br>□ Neutral.<br>□ Agree.<br>□ Strongly agree.
6. I would be willing to perform CPR even though administering CPR to a stranger may raise legal issues.	□ Strongly disagree.<br>□ Disagree.<br>□ Neutral.<br>□ Agree.<br>□ Strongly agree.
7. I am willing to perform CPR as appropriate for the specific circumstances, even if the patient may present with unsettling conditions such as vomiting or bleeding.	□ Strongly disagree.<br>□ Disagree.<br>□ Neutral.<br>□ Agree.<br>□ Strongly agree.
8. I am willing to perform CPR while taking proper personal protective measures (such as wearing a mask and performing only chest compressions), even if the patient may have a contagious disease.	□ Strongly disagree.<br>□ Disagree.<br>□ Neutral.<br>□ Agree.<br>□ Strongly agree.
**Section Six: Behavioural intention (Please mark "√" in the appropriate box for each item based on your own understanding. Only one answer is allowed for each item.)**	
If I had received CPR training, then as a bystander at the scene of an out-of-hospital cardiac arrest	
1. I would be willing to perform CPR to save my loved ones or friends.	□ Strongly disagree.<br>□ Disagree.

	□ Neutral.<br>□ Agree.<br>□ Strongly agree.
2. I would be willing to perform CPR to help someone I know.	□ Strongly disagree.<br>□ Disagree.<br>□ Neutral.<br>□ Agree.<br>□ Strongly agree.
3. I would be willing to perform CPR to help a stranger.	□ Strongly disagree.<br>□ Disagree.<br>□ Neutral.<br>□ Agree.<br>□ Strongly agree.
**The questionnaire is complete. Thank you once again for your support and assistance!**	
